# Early-life maternal probiotic supplementation programs sex- and region–specific gene expression in the adult offspring brain

**DOI:** 10.1016/j.bbih.2026.101191

**Published:** 2026-02-03

**Authors:** Tatiana Siegler Lathrop, Inés Martínez Sanchez, Ioannis S. Chronakis, Rochellys Diaz Heijtz

**Affiliations:** a Technical University of Denmark, DTU-Food, Research Group for Food Production Engineering, Laboratory of Nano-Bioscience, Kgs. Lyngby, Denmark; b Department of Neuroscience, Karolinska Institutet, Stockholm, Sweden

**Keywords:** Maternal microbiota, Probiotics, Hippocampus, Hypothalamus, Sex differences, Neuroimmune, Neuroplasticity

## Abstract

**Background:**

The maternal perinatal environment shapes brain development and long-term neurodevelopmental trajectories. Probiotic supplementation during this period has emerged as a promising strategy to support healthy neurodevelopmental outcomes through modulation of immune and synaptic plasticity pathways. However, the persistence and specificity of molecular effects in the offspring brain, particularly with respect to sex and brain region, remain poorly understood.

**Methods:**

We conducted two independent mouse experiments using different probiotic strains and exposure windows to evaluate the long-term transcriptional effects of maternal probiotic supplementation. Time-mated C57BL/6JRj dams received a multi-species probiotic (Ecologic® Panda) from gestational day (GD) 6 until birth, whereas BALB/cJRj dams received *Limosilactobacillus reuteri* (*L. reuteri*) from GD6 through postnatal day 7. The hippocampus and hypothalamus from adult male and female offspring were analyzed by RT-qPCR for genes related to synaptic plasticity, oxytocin signaling, neuroimmune regulation, myelination, and peptidoglycan (PGN) transport.

**Results:**

Multi-species supplementation induced broad and persistent transcriptional changes in hippocampus and hypothalamus, with generally larger effects in males. Altered transcripts included markers of synaptic plasticity (*Bdnf*, *Ppp1r1b*, *Syp*), immune regulation (*Il10, Trem2*), myelination (*Mag*, *Mog*), oxytocin signaling (*Oxtr*), and PGN transport (*Slc15a1*, *Slc15a2*, *Slc46a2*). In contrast, *L. reuteri* produced selective, sex- and region-dependent transcriptional effects that differed by brain region and sex. Notably, across probiotic conditions, *Il10* was consistently upregulated in both brain regions and sexes.

**Conclusions:**

These findings highlight that short, targeted maternal probiotic supplementation during the perinatal period is associated with persistent molecular signatures in the adult offspring brain across genetic backgrounds, converging on neuroimmune-related pathways.

## Highlights

• Early-life maternal probiotics program persistent brain gene expression in adult offspring.
• Maternal probiotics induce distinct hippocampal and hypothalamic transcriptional profiles.
• Multi-species probiotics exert broader transcriptional effects than *Limosilactobacillus reuteri*.
• Sex-specific responses reveal differential brain sensitivity in males and females.

## 1 Introduction

The gut–brain axis is increasingly recognized as a bidirectional communication network linking the gastrointestinal microbiota and the central nervous system (Cryan et al., 2020). During the perinatal period, microbiota-derived functional signals such as metabolites and peptidoglycan (PGN) fragments originating from the maternal microbiome can influence fetal and neonatal brain development, modulating circuit maturation (Martinez Sanchez et al., 2025; Vuong et al., 2020). Moreover, the developing fetal brain is particularly sensitive to inflammatory signals, as cytokines produced during immune activation can influence neurodevelopmental processes, including synapse formation, microglial maturation, and myelination (Bilbo and Schwarz, 2009; Fung et al., 2017). Accordingly, disruptions in early-life immune signaling, whether pro- or anti-inflammatory, can exert long-lasting consequences on neural circuit maturation and behavior.

Perturbations of the maternal microbiota arising from factors such as diet, stress, or antibiotic exposure have been associated with altered offspring neurodevelopmental outcomes (Bolte et al., 2022). Maternal inflammation is increasingly recognized as a key mediator linking maternal gut microbial states to fetal brain development. Elevated pro-inflammatory cytokines during pregnancy have been shown to alter fetal brain development and increase neurodevelopmental risk (Choi et al., 2016). Conversely, anti-inflammatory cytokines such as interleukin-10 (IL-10) play protective and organizational roles in shaping early neural circuits and maintaining microglial homeostasis (Fung et al., 2017). Both multi-species probiotic formulations and single-strain probiotics such as *Limosilactobacillus reuteri* (*L. reuteri*) have been shown to broadly modulate host inflammatory tone, in part through stimulation of the anti-inflammatory cytokine IL-10 (Luo et al., 2023; Niers et al., 2005). Thus, modulation of immune signaling through probiotic supplementation has emerged as a promising strategy to support healthy brain development, as several probiotic formulations reduce pro-inflammatory cytokines and are associated with improvements in cognitive or affective outcomes across preclinical and clinical studies (Kumar et al., 2024).

Recent conceptual work highlights that maternal microbiome-derived signals can shape offspring neurodevelopmental trajectories via cytokine signaling, microbial metabolites, and microglial programming (Orchanian and Hsiao, 2025). Furthermore, probiotic supplementation during early life has been shown to produce long-term neurodevelopmental effects in animal models; however, most studies extend exposure well into late postnatal life or focus primarily on behavioral outcomes (Cuinat et al., 2022). In contrast, the enduring molecular consequences of maternal probiotic intake confined to defined perinatal windows, including the extent to which such exposure programs persistent, sex- and brain region-specific transcriptional changes, are still poorly understood. We recently demonstrated that maternal supplementation with a multi-strain probiotic induces enduring, sex-dependent transcriptional effects in the offspring prefrontal cortex in adulthood (Siegler Lathrop et al., 2024). Similarly, *L. reuteri* supplementation influences transcriptional programs in both the prefrontal cortex and striatum of adult offspring (Siegler Lathrop et al., 2025). Whether similar molecular programming occurs in other brain regions remains unknown.

The hippocampus, essential for learning, memory, and stress regulation (Larosa and Wong, 2022; Pronier et al., 2023), and the hypothalamus, a central regulator of homeostasis and immune–neuroendocrine integration (Goel et al., 2025), are both developmentally plastic and sensitive to environmental cues. These regions are strongly shaped by microglial activity and oligodendrocyte maturation, processes that are increasingly recognized as being influenced by the gut microbiota (Cryan et al., 2020). Despite their relevance, few studies have examined whether maternal probiotic supplementation during targeted perinatal windows induces persistent transcriptional changes in the hippocampus and hypothalamus. Such changes may have implications for cognition, stress regulation, metabolism, and social behavior, potentially converging on shared immune-sensitive molecular pathways. Given that both excessive pro-inflammatory signaling and insufficient anti-inflammatory control during early life can disrupt brain development (Bilbo and Schwarz, 2009; Dinel et al., 2014), it is critical to determine whether maternal probiotic supplementation programs anti-inflammatory pathways, such as IL-10, in a region- and sex-specific manner.

Here, we tested the hypothesis that maternal probiotic supplementation during defined perinatal windows programs persistent, sex- and brain region–specific transcriptional signatures in neural circuits involved in memory, stress regulation, and neuroendocrine homeostasis. To address this, we examined the long-term transcriptional effects of maternal probiotic supplementation in two independent mouse experiments differing in probiotic strain and exposure window. The hippocampus and hypothalamus were selected due to their developmental plasticity and sensitivity to immune- and microbiota-derived signaling. In parallel experiments, C57BL/6JRj dams received a multi-strain probiotic formulation (Ecologic® Panda) from gestational day (GD) 6 until birth, whereas BALB/cJRj dams received *L. reuteri* from GD6 through postnatal day (P) 7. This design enabled identification of both shared and intervention-specific molecular pathways through which maternal probiotic exposure exerts enduring effects on hippocampal and hypothalamic gene expression.

## 2 Materials and methods

### 2.1 Animals

Time-mated pregnant C57BL/6JRj and BALB/cJRj dams were obtained from Janvier Labs (Le Genest-Saint-Isle, France) and housed individually under standard laboratory conditions at the Comparative Medicine–Biomedicum (KM-B), Karolinska Institutet. Dams were housed in Makrolon Type III polycarbonate cages under controlled conditions (22 °C, 55% ± 10% humidity) on a 12 h light–dark cycle, with bedding and nesting enrichment material. Each dam and her litter had ad libitum access to autoclaved water and standard chow. Offspring in each treatment group were derived from multiple litters, with both sexes represented in all groups. To minimize litter effects, each treatment group included no more than two pups per litter, with balanced sampling across litters. All procedures were approved by the Regional Ethics Committee on Animal Research, Stockholm North (Dnr 12837-2020), and were conducted in accordance with EU Directive 2010/63/EU. No exclusion criteria were pre-specified, and no animals were excluded or died during the study.

### 2.2 Probiotic treatment

Two independent animal experiments were conducted. In Experiment 1, C57BL/6JRj dams (n = 5 per treatment group) received a multi-species probiotic formulation (Ecologic® Panda, Winclove Probiotics B.V., Amsterdam, The Netherlands) containing *Bifidobacterium bifidum* W23, *B. lactis* W51, *B. lactis* W52, and *Lactococcus lactis* W58, or a matched control formulation (n = 5 dams), from gestational day (GD) 6 until birth, as previously described (Siegler Lathrop et al., 2024). In Experiment 2, BALB/cJRj dams (n = 5 per treatment group) received *Limosilactobacillus reuteri* W192 (batch 20G0138; formerly *Lactobacillus reuteri*) or control from GD6 until P7, extending exposure into early lactation, as previously described (Siegler Lathrop et al., 2025). The extended exposure window in Experiment 2 was chosen to encompass both prenatal and early postnatal maternal–offspring transfer during a critical period of hypothalamic and hippocampal circuit maturation. Both probiotic formulations were supplied in a rice starch/maltodextrin carrier matrix, which was also used for control treatments. Preparations were dissolved in sterile drinking water at a concentration of 8 × 10^7^ CFU/mL and replaced daily. Oral administration via drinking water was chosen to minimize handling stress compared with gavage. Pregnant dams were assigned to probiotic or control groups by the experimenter, and water intake was visually monitored daily by animal facility personnel, with no differences observed between groups.

### 2.3 Tissue collection and RNA extraction

A total of six adult offspring were analyzed per sex and treatment group (n = 6), resulting in 24 offspring per experiment. Animals were euthanized between 9 and 12 weeks of age by cervical dislocation between 09:00 and 11:00, with tissue collection occurring at 9 weeks for C57BL/6JRj offspring and between 11 and 12 weeks for BALB/cJRj offspring. The hippocampus and hypothalamus were rapidly dissected on ice, frozen on dry ice, and stored at −80 °C for no longer than one month prior to RNA extraction.

Total RNA was extracted using the RNeasy® Mini Kit (Qiagen, Cat. No. 74104) according to the manufacturer's instructions. Approximately 20–30 mg of tissue was used per sample. Tissue homogenization was performed using 5 mm stainless steel beads in a TissueLyser II (Qiagen). RNA concentration and purity were assessed using a NanoDrop 2000C spectrophotometer (Thermo Fisher Scientific). Extracted RNA was stored at −80 °C until further processing.

### 2.4 cDNA synthesis and quantitative RT-qPCR

cDNA was synthesized from 1000 ng of total RNA using the iScript™ cDNA Synthesis Kit (Bio-Rad) and stored at −20 °C. Quantitative real-time PCR was performed on a QuantStudio™ 7 Real-Time PCR System (Applied Biosystems) using SYBR Green chemistry. Primer efficiency was verified using standard curves, and product specificity was confirmed by single-peak melt curve analysis. Primer sequences are provided in Table S1. Gene symbols are reported according to mouse nomenclature (e.g., *Il10*) and refer to mRNA expression levels measured by RT-qPCR, whereas protein names (e.g., IL-10) are used only when referring to the protein levels.

Peptidylprolyl isomerase A (*Ppia*), hypoxanthine-guanine phosphoribosyltransferase (*Hprt*), and glyceraldehyde-3-phosphate dehydrogenase (*Gapdh*) were selected as reference genes based on prior validation of their expression stability in rodent tissues and on empirical assessment of their stability under the experimental conditions used here (Svingen et al., 2015). Relative gene expression was calculated using the ΔΔCt method, with normalization to the most stable reference gene(s) per plate. All samples were analyzed in technical duplicates.

### 2.5 Candidate gene selection and rationale

Candidate genes were selected *a priori* based on their established roles in neurodevelopment, social behavior, neuroimmune signaling, myelination, and microbiota–gut–brain communication. Building on our prior work and the broader literature on microbiota–brain interactions, we assessed the expression of a targeted panel of genes in the hippocampus and hypothalamus representing distinct functional domains relevant to early-life microbial modulation of brain development. Genes related to neuroplasticity and synaptic function included *Bdnf* (brain-derived neurotrophic factor), *Ppp1r1b* (DARPP-32), and *Syp* (synaptophysin), which are critical for synaptic maturation, plasticity, and neurotransmission. To capture neuroimmune and microglia-associated pathways, we examined *Itgam* (CD11b), *Il10* (interleukin-10), and *Trem2* (triggering receptor expressed on myeloid cells 2), genes implicated in immune regulation, microglial activation states, and neuroimmune crosstalk. Genes related to myelination and oligodendrocyte function included *Mag* (myelin-associated glycoprotein) and *Mog* (myelin oligodendrocyte glycoprotein), which reflect white matter development and integrity. In addition, we included *Oxtr* (oxytocin receptor) due to its established role in social behavior and neurodevelopmental outcomes, as well as its reported sensitivity to microbiota-dependent modulation (Sgritta et al., 2019). To directly probe potential mechanisms linking bacterial cell wall components to the brain, we quantified the expression of three peptidoglycan (PGN) transporters: *Slc15a1* (PepT1), *Slc15a2* (PepT2), and *Slc46a2*, which have been implicated in the cellular uptake and sensing of PGN-derived fragments (Bastos et al., 2021).

Together, this hypothesis-driven gene panel was designed to capture complementary aspects of synaptic plasticity, neuroimmune signaling, myelination, social behavior, and microbial-derived molecular transport, allowing us to assess how targeted early-life probiotic exposure shapes long-term transcriptional programs in key brain regions.

### 2.6 Statistical analysis

All statistical analyses were performed using GraphPad Prism (version 10). For each brain region (hippocampus or hypothalamus) and experimental condition (multi-species probiotic or *Limosilactobacillus reuteri* supplementation), log_2_ fold change values from independent biological samples were compared between probiotic-treated and control groups using unpaired two-tailed t-tests with Welch's correction. Welch's *t*-test was selected *a priori* as a conservative approach that does not assume equal variances between groups. To control for multiple comparisons, Benjamini–Hochberg false discovery rate (FDR) correction was applied across the full gene panel within each sex × brain region × treatment comparison. FDR-adjusted q values are reported in Table S2–S5, and genes with q < 0.05 were considered statistically significant after FDR correction. Analyses were conducted separately for males and females. Data are presented as mean ± SEM. In the figures, individual data points represent fold-change values for each biological sample, whereas statistical analyses were performed on the corresponding log_2_-transformed data. Statistical significance in figures is indicated as q < 0.05 (∗), q < 0.01 (∗∗), and q < 0.001 (∗∗∗). Exact P values, t statistics, degrees of freedom, and FDR-adjusted q values are provided in the supplementary tables. No statistical outliers were identified or excluded from any analysis.

## 3 Results

Detailed statistical results for each brain region, sex, and experimental condition, including exact P values, t statistics, degrees of freedom, and FDR-adjusted q values, are provided in Table S2–S5.

### 3.1 Prenatal multi-species probiotic exposure programs persistent sex-specific gene expression in the hippocampus and hypothalamus of adult offspring

#### 3.1.1 Hippocampus

**Fig. 1:**
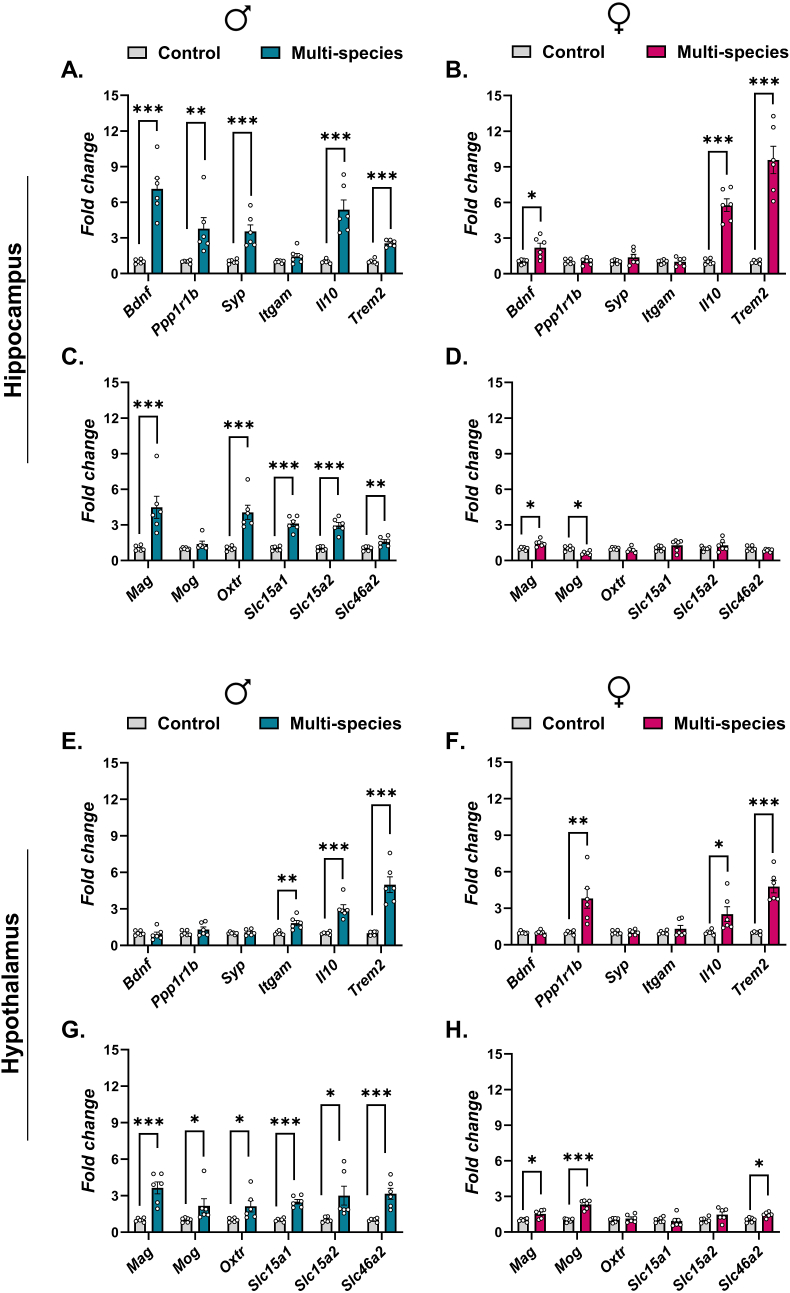
**Persistent transcriptional effects of maternal multi-species probiotic supplementation on adult offspring hippocampus and hypothalamus**. C57BL/6JRj dams received a multi-strain (Ecologic® Panda) from GD6 to birth. mRNA expression in hippocampus (A–D) and hypothalamus (E–H) was quantified in male (♂) and female (♀) offspring by RT-qPCR. Genes analyzed included markers of neurotrophic signaling (*Bdnf*), synaptic integrity (*Ppp1r1b*, *Syp*), immune regulation (*Itgam*, *Il10*, *Trem2*), myelination (*Mag*, *Mog*), oxytocin receptor (*Oxtr*), and solute carriers (*Slc15a1*, *Slc15a2*, *Slc46a2*). Data are shown as mean ± SEM with individual biological replicates plotted and are expressed as fold change relative to sex-matched controls. Statistical analyses were performed on log_2_-transformed fold-change values using unpaired two-tailed t-tests with Welch's correction. Multiple testing across the gene panel within each sex × brain region comparison was controlled using the Benjamini–Hochberg false discovery rate (FDR). Statistical significance is indicated as q < 0.05 (∗), q < 0.01 (∗∗), and q < 0.001 (∗∗∗).

In male offspring, prenatal exposure to the multi-species probiotic induced widespread and persistent transcriptional changes in the hippocampus relative to sex-matched controls (Fig. 1A and C; Table S2). Genes associated with neuroplasticity and neurotransmission, including *Bdnf*, *Ppp1r1b*, and *Syp*, were significantly upregulated following FDR correction (q < 0.05). In parallel, neuroimmune-related genes *Il10* and *Trem2* were also significantly upregulated, whereas *Itgam* was not significantly altered. In addition, genes related to myelination, oxytocin signaling, and microbial–host signaling, including *Mag*, *Oxtr,* and the PGN transporters *Slc15a1*, *Slc15a2*, and *Slc46a2*, were significantly upregulated relative to controls (q < 0.05), whereas *Mog* remained unchanged.

In contrast, female offspring displayed a more selective hippocampal transcriptional response to prenatal multi-species probiotic exposure (Fig. 1B and D; Table S2). Significant upregulation relative to controls was observed for *Bdnf*, *Il10*, and *Trem2* after FDR correction (q < 0.05), whereas synaptic genes (*Ppp1r1b*, *Syp*) and the immune cell marker *Itgam* were not significantly altered. Among myelination-related genes, *Mag* was significantly upregulated, whereas *Mog* was significantly downregulated. In contrast, *Oxtr* and PGN transporter genes (*Slc15a1*, *Slc15a2*, and *Slc46a2*) remained unchanged relative to controls.

#### 3.1.2 Hypothalamus

In male offspring, prenatal exposure to the multi-species probiotic induced broad transcriptional upregulation in the hypothalamus relative to controls (Fig. 1E and G; Table S3). Genes associated with neuroimmune and microglial signaling, including *Itgam*, *Il10,* and *Trem2*, were significantly upregulated following FDR correction (q < 0.05). In addition, significant upregulation relative to controls was observed for myelination-related genes (*Mag* and *Mog*), oxytocin signaling (*Oxtr*), and all three PGN transporters (*Slc15a1*, *Slc15a2*, and *Slc46a2*).

In contrast, female offspring exhibited a more selective hypothalamic transcriptional response to prenatal multi-species probiotic exposure (Fig. 1F and H; Table S3). Significant upregulation relative to controls was detected for *Ppp1r1b*, *Il10*, and T*rem2* (q < 0.05), whereas *Bdnf*, *Syp*, and *Itgam* were not significantly altered. Among genes related to myelination and microbial–host signaling, *Mag*, *Mog*, and *Slc46a2* were significantly upregulated, while *Oxtr*, S*lc15a1*, and *Slc15a2* remained unchanged relative to controls.

### 3.2 Perinatal *L. reuteri* exposure programs sex-specific transcriptional changes in the hippocampus and hypothalamus of adult offspring

#### 3.2.1 Hippocampus

**Fig. 2:**
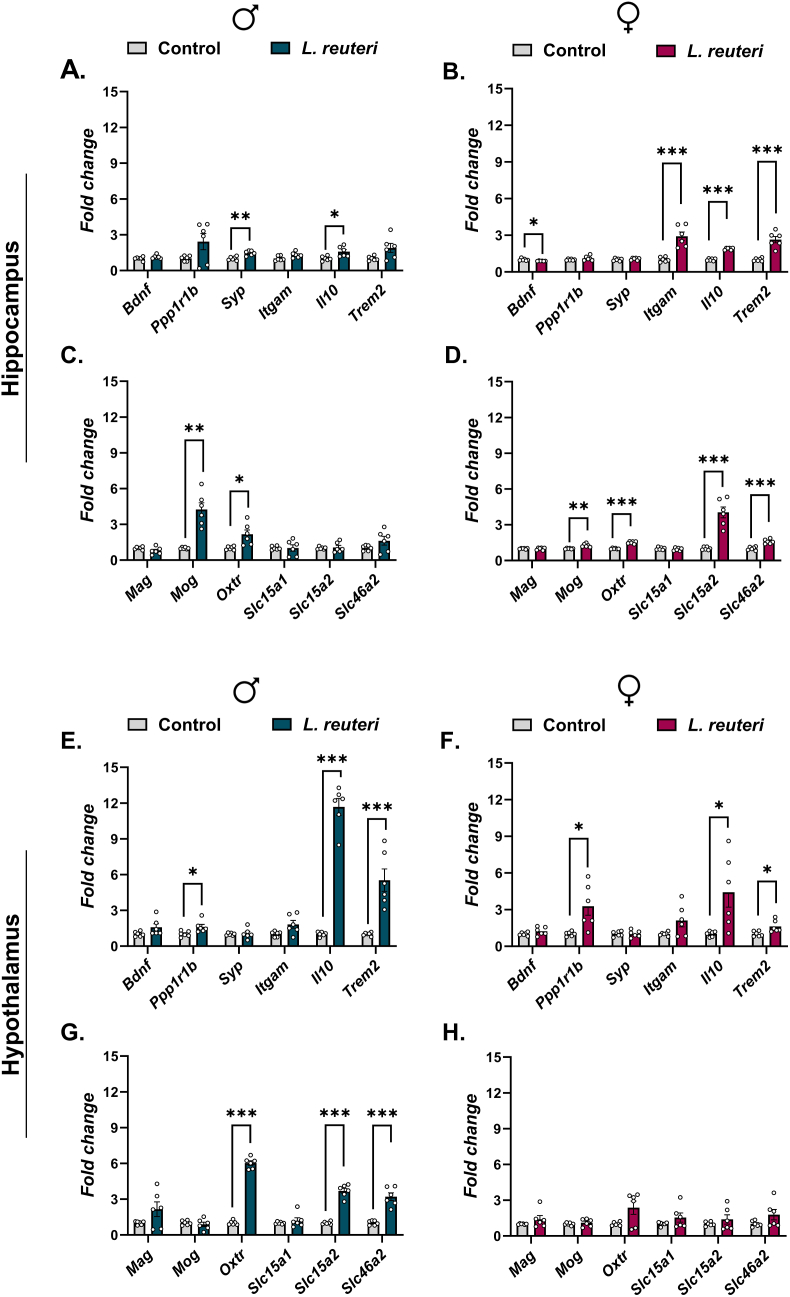
**Persistent transcriptional effects of maternal *Limosilactobacillus reuteri* supplementation on adult offspring hippocampus and hypothalamus**. BALB/c dams received *L. reuteri* from GD6 to P7. mRNA expression in hippocampus (A–D) and hypothalamus (E–H) was quantified in male (♂) and female (♀) offspring by RT-qPCR. Genes analyzed included markers of neurotrophic signaling (*Bdnf*), synaptic integrity (*Ppp1r1b*, *Syp*), immune regulation (*Itgam*, *Il10*, *Trem2*), myelination (*Mag*, *Mog*), oxytocin receptor (*Oxtr*), and solute carriers (*Slc15a1*, *Slc15a2*, *Slc46a2*). Data are shown as mean ± SEM with individual biological replicates plotted and are expressed as fold change relative to sex-matched controls. Statistical analyses were conducted on log_2_-transformed fold-change values using unpaired two-tailed t-tests with Welch's correction. Multiple testing across the gene panel within each sex × brain region comparison was controlled using the Benjamini–Hochberg FDR procedure. Statistical significance is indicated as q < 0.05 (∗), q < 0.01 (∗∗), and q < 0.001 (∗∗∗).

In male offspring, perinatal exposure to *L. reuteri* induced a selective hippocampal transcriptional response relative to controls (Fig. 2A and C; Table S4). Significant upregulation after FDR correction (*q* < 0.05) was detected for *Syp* and *Il10*, whereas *Bdnf*, *Ppp1r1b*, *Itgam*, and *Trem2* were not significantly altered. Among myelination- and oxytocin-related genes, *Mog* and *Oxtr* were significantly upregulated (q < 0.05), whereas *Mag* and PGN transporter genes (*Slc15a1*, *Slc15a2*, *Slc46a2*) remained unchanged relative to controls.

In female offspring, perinatal *L. reuteri* exposure resulted in hippocampal transcriptional changes (Fig. 2B and D; Table S4). Significant upregulation relative to controls was observed for *Itgam*, *Il10*, and *Trem2* (q < 0.05), whereas *Bdnf* showed a modest but significant downregulation after FDR correction. In addition, genes associated with oxytocin signaling and microbial sensing, including *Oxtr*, *Slc15a2*, and *Slc46a2*, were significantly upregulated, along with the myelination-related gene *Mog*. Other synaptic genes (*Ppp1r1b, Syp*) and *Slc15a1* were not significantly altered.

#### 3.2.2 Hypothalamus

In the male hypothalamus, perinatal *L. reuteri* exposure produced significant transcriptional upregulation across a subset of genes relative to controls (Fig. 2E and G; Table S5). Significant increases were detected for the anti-inflammatory cytokine *Il10* and the microglia-associated receptor *Trem2* (q < 0.05), along with the synaptic-related gene *Ppp1r1b*. In addition, genes related to oxytocin signaling (*Oxtr*) and PGN transport (*Slc15a2* and *Slc46a2*) were significantly upregulated. In contrast, *Bdnf, Syp*, *Itgam*, *Mag*, *Mog*, and *Slc15a1* were not significantly altered.

In female offspring, hypothalamic transcriptional changes following perinatal *L. reuteri* exposure were restricted (Fig. 2F and H; Table S5). Significant upregulation relative to controls was observed for *Ppp1r1b*, *Il10*, and *Trem2* (q < 0.05), while genes related to synaptic plasticity (*Bdnf*, *Syp*), myelination (*Mag*, *Mog*), oxytocin signaling (*Oxtr*), and PGN transport (*Slc15a1*, *Slc15a2*, *Slc46a2*) were not significantly altered.

### 3.3 Summary of probiotic-induced transcriptional patterns

**Fig. 3:**
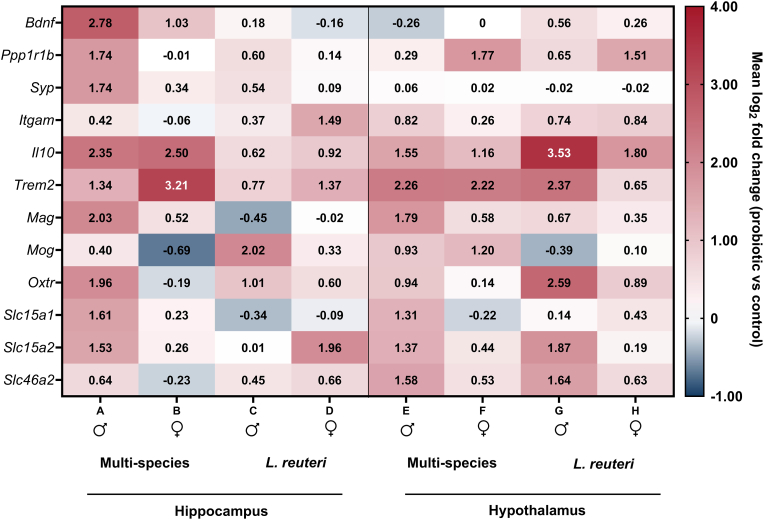
**Heatmap of hippocampal and hypothalamic gene expression in adult offspring after maternal probiotic supplementation**. Heatmaps display mean log2 fold change (probiotic vs control) for males (♂) and females (♀). Dams received either a multi-species probiotic formulation (Ecologic® Panda) from gestational day (GD) 6 until birth, or *Limosilactobacillus reuteri* (*L. reuteri*) from GD6 to postnatal day (P) 7. Gene expression was assessed in the hippocampus and hypothalamus of adult offspring. Red indicates increased expression, and blue indicates decreased expression relative to control (log_2_ fold change), with white representing no change (log_2_ fold change = 0). (For interpretation of the references to colour in this figure legend, the reader is referred to the Web version of this article.)

To facilitate integrative interpretation of the gene-wise analyses, Fig. 3 provides a heatmap summary of mean log_2_ fold changes in hippocampal and hypothalamic gene expression following targeted early-life probiotic exposure. The heatmap visualizes the direction and magnitude of transcriptional changes across sex, brain region, and probiotic condition, without conveying statistical significance, which is instead reported in Fig. 1, Fig. 2 and Table S2–S5. Consistent with the gene-level analyses, multi-species probiotic exposure induced broader transcriptional modulation across multiple functional domains, particularly in males, whereas *L. reuteri* exposure produced a more selective, sex- and region-dependent profile. Across probiotic conditions and brain regions, *Il10* and *Trem2* showed consistent upregulation relative to controls, and coordinated changes in PGN transporter genes (*Slc15a1, Slc15a2,* and *Slc46a2*) were evident.

## 4 Discussion

The maternal microbiota critically shapes offspring neurodevelopment, and targeted probiotic modulation during early developmental windows has emerged as a promising strategy to support healthy brain development (Cuinat et al., 2022). Here, we show that maternal supplementation with either a multi-species probiotic during pregnancy (GD6–birth) or *Limosilactobacillus reuteri* from GD6–P7 is associated with persistent, region-specific, and sex-dependent transcriptional changes in the adult offspring brain. Across the hippocampus and hypothalamus, these changes involved genes related to neuroplasticity, neuroimmune regulation, myelination, oxytocin signaling, and pathways linked to bacterial PGN sensing and transport. Together, these findings indicate that maternal probiotic exposure during short, targeted perinatal windows can shape long-term molecular programs in offspring brain regions implicated in cognition, stress regulation, and neuroendocrine function.

While both probiotic interventions converged on persistent transcriptional changes in the hippocampus and hypothalamus, the scope and pattern of effects differed. The multi-species probiotic elicited broad transcriptional modulation, particularly in males, with coordinated upregulation of genes related to plasticity, myelination, and immune regulation. In contrast, *L. reuteri* supplementation produced a more selective profile, characterized by consistent modulation of *Oxtr* and *Il10*, with region- and sex-dependent effects on *Trem2*, pointing to a more focused engagement of anti-inflammatory and oxytocin-related signaling pathways. Collectively, these findings underscore the importance of probiotic composition for determining both the magnitude and specificity of neurodevelopmental programming.

A consistent finding across brain regions and probiotic formulations was the long-term modulation of PGN transporter expression, suggesting that PGN-related signaling may represent a common pathway through which the maternal microbiota influences neurodevelopmental outcomes. In our previous study using the same multi-species probiotic during pregnancy, we observed persistent upregulation of *Slc15a1* in the PFC of adult male offspring (Siegler Lathrop et al., 2024). More recently, we found increased expression of the PGN transporters *Slc46a2* and *Slc46a3* in the striatum of females, and of *Slc46a2* in the striatum of males, following perinatal *L. reuteri* exposure (Siegler Lathrop et al., 2025). Although these transporters can mediate uptake of a broader range of peptide substrates, their regulation in this context supports altered sensitivity to microbiota-derived cell wall components, potentially via immune-mediated mechanisms. The observation that these changes persist into adulthood indicates that short perinatal probiotic exposures, whether multi-species or single-strain, can durably shape molecular programs through which neural circuits sense and respond to microbiota-derived molecules. Given emerging links between PGN signaling and neurodevelopment (Gonzalez-Santana and Diaz Heijtz, 2020), these observations highlight a potentially unifying mechanism and translational target for microbiota-based interventions.

The hippocampus and hypothalamus exhibited distinct transcriptional signatures. The hippocampus, central to learning, memory, and stress regulation (Larosa and Wong, 2022; Pronier et al., 2023), showed pronounced modulation of plasticity-related transcripts (*Bdnf*, *Ppp1r1b, Syp*), particularly in males prenatally exposed to the multi-species probiotic. In contrast, the hypothalamus, a key regulator of homeostatic and neuroendocrine processes (Goel et al., 2025), showed stronger modulation of immune (*Il10*, *Trem2*) and oxytocin-related transcripts (*Oxtr*). Interestingly, myelin-associated transcripts also diverged across regions: in females, *Mag* increased in both hippocampus and hypothalamus, whereas *Mog* decreased in the hippocampus but increased in the hypothalamus. This region-specific directionality suggests that maternal probiotics may differentially shape oligodendrocyte biology depending on local circuit environments. These findings align with our previous studies in the PFC using the same probiotic formulations and developmental windows, further supporting the view that brain regions differ in their susceptibility to microbiota-driven programming (Siegler Lathrop et al., 2024, 2025). Such regional specificity may contribute to differential modulation of cognitive versus homeostatic and neuroendocrine functions, depending on the circuitry involved.

The observed modulation of myelin-related genes (*Mag*, *Mog*) across the hippocampus and hypothalamus suggests region-specific effects on oligodendrocyte-associated processes with potential implications for axon–glia interactions and circuit organization. These transcriptional changes resonate with accumulating evidence that the gut microbiota influences oligodendrocyte biology and myelination (Sauma and Casaccia, 2020). For example, germ-free mice exhibit altered myelin gene expression and ultrastructure in the prefrontal cortex, which can be normalized following microbial colonization (Hoban et al., 2016). Notably, Needham et al. (2022) demonstrated that the microbial metabolite 4-ethylphenyl sulfate impairs oligodendrocyte maturation and reduces myelination, linking gut-derived signals directly to white matter abnormalities and behavioral alterations (Needham et al., 2022). Although direct evidence for probiotic-induced effects on myelination is still limited, maternal supplementation with *Lactobacillus* and *Bifidobacterium* mixtures has been shown to influence oligodendrocyte progenitor development in offspring (Lu et al., 2020). Together, these findings support the hypothesis that probiotics may indirectly regulate myelination via microbiota-derived metabolites, immune signaling, or both. Future studies should therefore determine whether maternal probiotic interventions influence oligodendrocyte maturation and white matter connectivity, and how such molecular changes relate to long-term functional outcomes.

Sex emerged as an important determinant of transcriptional outcomes. Males generally showed broader modulation, including hippocampal increases in *Ppp1r1b*, *Oxtr,* and *Trem2*, together with hypothalamic upregulation of *Il10*, *Trem2,* and PGN transporter genes (*Slc15a1*, *Slc15a2*, and *Slc46a2*). Females exhibited more selective immune-related changes in both hippocampus and hypothalamus. Notably, *Trem2* was significantly increased across sexes in both the hippocampus and hypothalamus following multi-species exposure, whereas after *L. reuteri* exposure *Trem2* upregulation was strongest in the hypothalamus and more evident in females.

Similarly, *Il10* expression increased across sexes in both hippocampus and hypothalamus following multi-species supplementation and was also upregulated in both regions after *L. reuteri* exposure. Such sexual dimorphism, alongside shared signatures, likely reflects the interplay of sex hormones, microglial maturation, and microbiota-derived signaling, with implications for sex-specific neurodevelopmental trajectories (Bobotis et al., 2023; Jasarevic et al., 2016).

The upregulation of *Il10* observed across brain regions is notable given the central role of *IL-10* in maintaining neuroimmune homeostasis. *IL-10* modulates microglial activation thresholds, limits excessive inflammatory signaling, and preserves synaptic function (Lobo-Silva et al., 2016). Beyond its canonical anti-inflammatory role, *IL-10* can directly promote synapse formation in hippocampal neurons, suggesting that sustained *Il10* upregulation may influence both immune tone and synaptic remodeling in offspring brain circuits (Lim et al., 2013). Together, these findings suggest that *Trem2* and *Il10* may represent partially shared neuroimmune targets of maternal probiotic supplementation. Consistent with this interpretation, adult probiotic supplementation has been shown to increase brain *Il10 expression* and improve hippocampal spine density and cognitive performance in a transgenic mouse model of Alzheimer's disease (Webberley et al., 2022), supporting a broader role for probiotic-associated *Il10* modulation in shaping synaptic function and behavioral outcomes.

Several limitations should be acknowledged. Behavioral outcomes were not assessed in the present cohort; however, in prior studies using comparable probiotic paradigms, prenatal exposure to the same multi-species probiotic reduced anxiety-like behavior in male offspring, whereas perinatal *L. reuteri* supplementation selectively enhanced social recognition without major effects on anxiety-related behavior (Siegler Lathrop et al., 2024, 2025). Gut microbiota composition was also not assessed here; nevertheless, we previously reported long-lasting alterations in adult gut microbiota following both interventions, including increased abundance of short-chain fatty acid–producing taxa, particularly in males. Because the two experiments were conducted in distinct mouse strains, inherent differences in baseline immune responsiveness may have contributed to variability in transcriptional outcomes. We did not directly quantify microbiota-derived metabolites or PGN levels in the brain, nor did we assess blood–brain barrier integrity, which limits mechanistic inference regarding the molecular intermediates linking maternal probiotic exposure to long-term transcriptional changes. Finally, the targeted candidate gene approach does not capture genome-wide or cell-type–specific transcriptional dynamics. Ongoing studies are addressing these limitations by integrating complementary experimental approaches.

In summary, our study demonstrates that maternal probiotic supplementation during critical perinatal windows programs persistent, sex-specific transcriptional signatures across multiple brain regions implicated in cognition, neuroendocrine regulation, and neuroimmune signaling. By comparing two probiotic interventions that differed in strain composition and exposure window, we identify both shared and intervention-specific molecular pathways, including convergence on neuroimmune regulators such as *Trem2* and *Il10*. These findings support the concept that early-life microbial modulation can exert long-lasting effects on brain molecular architecture in a region- and sex-dependent manner. Together, this work provides a framework for understanding how targeted maternal probiotic interventions may shape neurodevelopmental trajectories and inform future efforts to optimize microbiota-based strategies during defined sensitive developmental periods.

## CRediT authorship contribution statement

**Tatiana Siegler Lathrop:** Writing – review & editing, Writing – original draft, Project administration, Investigation, Formal analysis, Conceptualization. **Inés Martínez Sanchez:** Writing – review & editing, Investigation. **Ioannis S. Chronakis:** Writing – review & editing, Visualization, Supervision, Funding acquisition, Conceptualization. **Rochellys Diaz Heijtz:** Writing – review & editing, Writing – original draft, Supervision, Investigation, Funding acquisition, Conceptualization.

## Funding

This work was supported by funding from the Olle Enqvist Foundation (226-0123), the Swedish Medical Council (2018-06232), 10.13039/100012774IFD (7076-00053B), and 10.13039/501100005192DTU (Alliance-Stipendium).

## Declaration of competing interest

The authors declare that they have no known competing financial interests or personal relationships that could have appeared to influence the work reported in this paper.

## Data Availability

Data will be made available on request.
