# Supplementary material for: Early-life maternal probiotic supplementation programs sex- and region–specific gene expression in the adult offspring brain
Source: Brain Behav Immun Health. 2026 Feb 3;52:101191. doi: 10.1016/j.bbih.2026.101191 (PMC12906190; doi:10.1016/j.bbih.2026.101191)
Supplement: Multimedia component 1 [file mmc1.pdf]

Table S1. Primer sequences used for gene expression analysis

Gene	Forward primer (5'→3')	Reverse primer (5'→3')	Accession no.
Ppia	agcatacaggtcctggcatc	ttcaccttcccaaagaccac	NM_008907.2
Hprt	aagacttgctcgagatgtcatgaa	atccagcaggtcagcaaagaa	NM_013556.2
Gapdh	agcttgtcatcaacgggaag	tttgatgttagtggggtctcg	NM_001289726.1
Bdnf	gaagttggcttcctagcggt	taggccatgttgccttgtcc	NM_001048139.1
Oxtr	ctccacctacctgctgttgg	ttgacactactgacccgtgc	NM_001081147.1
Ppp1r1b	agcacctgcagaccattagc	ttccatctctctggggctca	NM_144828.1
Syp	tcacaggcactaccaacgtc	atctacaggtctgtggggct	NM_009305.2
Mag	gatgccctcgaccatctcag	gtgggcttccaaggtgcata	NM_010758.2
Mog	agttggggatgaagcagagc	gcaccgaagtcttccctctc	NM_010814.2
Il10	tgctgcctgctcttactgac	ttctgggccatgcttctctg	NM_010548.2
Itgam	aagcagctgaatgggaggac	tagatgcgatggtgtcgagc	NM_008401.2
Trem2	atgacacccttgctggaacc	gctagaggtgacccacagga	NM_001272078.2
Slc15a1	ggctcgattctacacctacatc	tgcccttcacatatttgtctgt	NM_053079.2
Slc15a2	ctagtccccctttcactgtag	gcgtttcctttggactcatttt	NM_021301.4
Slc46a2	tcgctggtggagtatcagga	gagtaagagtccgatgcggg	NM_021053.4
