# Supplementary material for: Early-life maternal probiotic supplementation programs sex- and region–specific gene expression in the adult offspring brain
Source: Brain Behav Immun Health. 2026 Feb 3;52:101191. doi: 10.1016/j.bbih.2026.101191 (PMC12906190; doi:10.1016/j.bbih.2026.101191)
Supplement: Multimedia component 2 [file mmc2.pdf]

Table S2. Differential hippocampal gene expression after multi-species probiotic supplementation

Males

Genes	Discovery?	P value	Mean of Control	Mean of Multi-species	Difference	SE of difference	t ratio	df	q value
Bdnf	Yes	0.000001	-0.001077	2.776	-2.777	0.2112	13.15	7.771	0.00001
Ppp1r1b	Yes	0.00168	-0.00198	1.743	-1.745	0.3099	5.633	5.608	0.00224
Syp	Yes	0.000147	-0.0006997	1.739	-1.74	0.2371	7.34	7.099	0.000252
Itgam	No	0.146868	0.0002896	0.4175	-0.4172	0.2486	1.678	5.705	0.146868
Il10	Yes	0.000027	-0.001729	2.345	-2.347	0.2303	10.19	6.637	0.000054
Trem2	Yes	0.000003	0.002039	1.338	-1.336	0.1217	10.98	8.234	0.000013
Mag	Yes	0.000229	0.001292	2.034	-2.033	0.2909	6.987	6.897	0.000344
Mog	No	0.093969	0.0006278	0.4042	-0.4036	0.2005	2.012	5.631	0.102511
Oxtr	Yes	0.000017	0.000727	1.956	-1.955	0.2171	9.002	8.112	0.000041
Slc15a1	Yes	0.000002	-0.00023	1.614	-1.614	0.1606	10.05	9.827	0.00001
Slc15a2	Yes	0.000006	-0.001107	1.533	-1.534	0.1728	8.876	9.592	0.000019
Slc46a2	Yes	0.006957	0.001026	0.6363	-0.6353	0.1842	3.449	9.287	0.008348

Females

Genes	Discovery?	P value	Mean of Control	Mean of Multi-species	Difference	SE of difference	t ratio	df	q value
Bdnf	Yes	0.008969	-0.0007465	1.027	-1.028	0.2883	3.565	7.091	0.021526
Ppp1r1b	No	0.976354	0.0001525	-0.006204	0.006357	0.2084	0.0305	8.769	0.976354
Syp	No	0.278153	-0.001223	0.3431	-0.3443	0.2906	1.185	6.415	0.41723
Itgam	No	0.801047	-0.0003808	-0.06378	0.0634	0.2422	0.2617	7.024	0.873869
Il10	Yes	<0.000001	-0.003576	2.497	-2.501	0.1892	13.22	9.973	0.000001
Trem2	Yes	<0.000001	0.0004867	3.206	-3.205	0.2002	16.01	7.374	0.000003
Mag	Yes	0.003666	-0.001256	0.521	-0.5222	0.1316	3.969	8.484	0.010998
Mog	Yes	0.00252	-0.001347	-0.6888	0.6875	0.1705	4.032	9.743	0.010079
Oxtr	No	0.247072	-0.001019	-0.192	0.191	0.1506	1.268	6.691	0.41723
Slc15a1	No	0.501713	0.001438	0.2333	-0.2319	0.3267	0.7098	6.715	0.602056
Slc15a2	No	0.33785	0.0009778	0.2638	-0.2628	0.2553	1.029	6.937	0.450466
Slc46a2	No	0.135869	0.001374	-0.2337	0.2351	0.143	1.644	8.682	0.271739

Statistical analyses were performed separately for males and females. Exact P values and Benjamini–Hochberg FDR-adjusted q values are reported.
Discovery indicates genes remaining significant after Benjamini–Hochberg FDR correction applied across the full gene panel within each experimental comparison.
Difference was calculated as Control − Probiotic; negative values indicate higher expression in the probiotic group.
