# Supplementary material for: Early-life maternal probiotic supplementation programs sex- and region–specific gene expression in the adult offspring brain
Source: Brain Behav Immun Health. 2026 Feb 3;52:101191. doi: 10.1016/j.bbih.2026.101191 (PMC12906190; doi:10.1016/j.bbih.2026.101191)
Supplement: Multimedia component 3 [file mmc3.pdf]

Table S3. Differential hypothalamic gene expression after multi-species probiotic supplementation

Males

Genes	Discovery?	P value	Mean of Control	Mean of Multi-species	Difference	SE of difference	t ratio	df	q value
Bdnf	No	0.399464	-0.002337	-0.2622	0.2599	0.2882	0.9017	6.482	0.435779
Ppp1r1b	No	0.277226	-0.002642	0.2935	-0.2961	0.2521	1.175	7.261	0.332671
Syp	No	0.687317	-0.0005033	0.05897	-0.05948	0.1415	0.4203	6.757	0.687317
Itgam	Yes	0.002351	-0.001088	0.8241	-0.8252	0.1796	4.595	7.168	0.004703
Il10	Yes	0.000035	0.000841	1.551	-1.55	0.1586	9.772	6.64	0.00014
Trem2	Yes	0.000006	-0.0003981	2.261	-2.261	0.2026	11.16	7.55	0.000035
Mag	Yes	0.000182	-0.0005188	1.787	-1.788	0.2412	7.413	6.705	0.000547
Mog	Yes	0.025124	-0.0008615	0.9301	-0.9309	0.3187	2.921	6.302	0.033499
Oxtr	Yes	0.018754	-0.0003924	0.9422	-0.9426	0.2964	3.18	6.074	0.028131
Slc15a1	Yes	0.000003	0.0001359	1.306	-1.306	0.1348	9.691	9.471	0.000035
Slc15a2	Yes	0.008324	0.001527	1.372	-1.37	0.3595	3.812	6.196	0.014269
Slc46a2	Yes	0.000317	0.002441	1.584	-1.582	0.2294	6.895	6.534	0.00076

Females

Genes	Discovery?	P value	Mean of Control	Mean of Multi-species	Difference	SE of difference	t ratio	df	q value
Bdnf	No	0.979402	-0.0009997	-0.00416	0.003161	0.1185	0.02666	7.736	0.979402
Ppp1r1b	Yes	0.001473	0.0001944	1.772	-1.771	0.3171	5.586	5.907	0.005894
Syp	No	0.908067	-0.002071	0.01567	-0.01774	0.1494	0.1187	9.232	0.979402
Itgam	No	0.410751	-0.001588	0.2556	-0.2572	0.2903	0.8861	5.804	0.605338
Il10	Yes	0.011763	-0.0008521	1.157	-1.158	0.3277	3.535	6.163	0.023526
Trem2	Yes	0.000002	-0.003387	2.219	-2.223	0.1645	13.51	7.119	0.00003
Mag	Yes	0.008653	0.001638	0.5816	-0.5799	0.1657	3.5	7.662	0.020768
Mog	Yes	0.000006	-0.001012	1.202	-1.203	0.1365	8.816	9.74	0.000036
Oxtr	No	0.504449	0.0004492	0.1405	-0.14	0.2012	0.6959	8.788	0.605338
Slc15a1	No	0.485207	-0.0008821	-0.2185	0.2176	0.2955	0.7364	7.062	0.605338
Slc15a2	No	0.185204	-0.004121	0.4427	-0.4468	0.305	1.465	7.196	0.317493
Slc46a2	Yes	0.005909	0.002496	0.5335	-0.531	0.1501	3.538	9.411	0.017727

Statistical analyses were performed separately for males and females. Exact P values and Benjamini–Hochberg FDR-adjusted q values are reported.
Discovery indicates genes remaining significant after Benjamini–Hochberg FDR correction applied across the full gene panel within each experimental comparison.
Difference was calculated as Control − Probiotic; negative values indicate higher expression in the probiotic group.
