# Supplementary material for: Early-life maternal probiotic supplementation programs sex- and region–specific gene expression in the adult offspring brain
Source: Brain Behav Immun Health. 2026 Feb 3;52:101191. doi: 10.1016/j.bbih.2026.101191 (PMC12906190; doi:10.1016/j.bbih.2026.101191)
Supplement: Multimedia component 4 [file mmc4.pdf]

Table S4. Differential hippocampal gene expression after *L. reuteri* probiotic supplementation

Males

Genes	Discovery?	P value	Mean of Control	Mean of *L. reuteri*	Difference	SE of difference	t ratio	df	q value
Bdnf	No	0.159788	-0.0001994	0.1832	-0.1834	0.1203	1.525	9.501	0.239682
Ppp1r1b	No	0.482951	0.003992	0.5977	-0.5937	0.7873	0.7542	5.291	0.579541
Syp	Yes	0.001512	0.001965	0.5409	-0.5389	0.1242	4.338	9.881	0.009072
Itgam	No	0.038972	0.001195	0.3668	-0.3656	0.1538	2.377	9.936	0.093532
Il10	Yes	0.010077	0.0008197	0.6217	-0.6209	0.1911	3.249	8.962	0.03023
Trem2	No	0.049056	-0.0003019	0.7726	-0.7729	0.3168	2.44	6.217	0.098112
Mag	No	0.104982	0.001029	-0.4523	0.4533	0.2374	1.909	5.976	0.179969
Mog	Yes	0.000087	-0.0009761	2.023	-2.024	0.1984	10.2	5.515	0.001046
Oxtr	Yes	0.007611	0.0004281	1.01	-1.009	0.2617	3.857	6.312	0.03023
Slc15a1	No	0.53557	0.000697	-0.3405	0.3412	0.5152	0.6622	5.301	0.584258
Slc15a2	No	0.980566	0.0003439	0.006175	-0.005831	0.2293	0.02543	5.792	0.980566
Slc46a2	No	0.338918	0.002121	0.4521	-0.45	0.4337	1.038	6.085	0.451891

Females

Genes	Discovery?	P value	Mean of Control	Mean of *L. reuteri*	Difference	SE of difference	t ratio	df	q value
Bdnf	Yes	0.028989	-0.00009173	-0.1576	0.1576	0.054	2.918	5.594	0.043484
Ppp1r1b	No	0.199402	-0.002253	0.1431	-0.1453	0.1016	1.431	6.402	0.26587
Syp	No	0.313561	-0.00173	0.08576	-0.08749	0.08238	1.062	9.861	0.376273
Itgam	Yes	0.000075	-0.001107	1.49	-1.491	0.2107	7.076	8.544	0.000181
Il10	Yes	0.000001	-0.0009612	0.9208	-0.9218	0.06433	14.33	7.255	0.000008
Trem2	Yes	0.000066	-0.001485	1.365	-1.366	0.165	8.28	7.12	0.000181
Mag	No	0.77573	-0.001059	-0.02135	0.02029	0.06832	0.297	6.492	0.77573
Mog	Yes	0.002151	0.001051	0.3302	-0.3291	0.0691	4.763	6.88	0.003687
Oxtr	Yes	<0.000001	-0.001347	0.5967	-0.598	0.0481	12.43	8.996	0.000007
Slc15a1	No	0.389514	-0.001896	-0.08835	0.08646	0.09599	0.9007	9.727	0.424924
Slc15a2	Yes	0.000024	0.0004258	1.963	-1.962	0.1892	10.37	6.661	0.000095
Slc46a2	Yes	0.000173	0.00156	0.6592	-0.6577	0.1106	5.947	9.515	0.000346

Statistical analyses were performed separately for males and females. Exact P values and Benjamini–Hochberg FDR-adjusted q values are reported.
Discovery indicates genes remaining significant after Benjamini–Hochberg FDR correction applied across the full gene panel within each experimental comparison.
Difference was calculated as Control − Probiotic; negative values indicate higher expression in the probiotic group.
