# Supplementary material for: Early-life maternal probiotic supplementation programs sex- and region–specific gene expression in the adult offspring brain
Source: Brain Behav Immun Health. 2026 Feb 3;52:101191. doi: 10.1016/j.bbih.2026.101191 (PMC12906190; doi:10.1016/j.bbih.2026.101191)
Supplement: Multimedia component 5 [file mmc5.pdf]

Table S5. Differential hypothalamic gene expression after *L. reuteri* probiotic supplementation

Males

Genes	Discovery?	P value	Mean of Control	Mean of *L. reuteri*	Difference	SE of difference	t ratio	df	q value
Bdnf	No	0.069424	-0.002752	0.5592	-0.5619	0.2646	2.124	7.35	0.104137
Ppp1r1b	Yes	0.012028	-0.002175	0.6494	-0.6516	0.212	3.073	9.82	0.024056
Syp	No	0.934879	0.0009096	-0.02086	0.02177	0.2548	0.08545	5.59	0.934879
Itgam	No	0.054925	-0.0007907	0.7358	-0.7365	0.3215	2.291	7.155	0.094157
Il10	Yes	<0.000001	-0.0009139	3.531	-3.532	0.1515	23.32	9.63	<0.000001
Trem2	Yes	0.000069	-0.001657	2.365	-2.367	0.2591	9.134	6.354	0.000166
Mag	No	0.292762	0.0008522	0.6657	-0.6648	0.5688	1.169	5.256	0.390349
Mog	No	0.377265	-0.001494	-0.3864	0.3849	0.4026	0.956	5.791	0.452718
Oxtr	Yes	<0.000001	0.002513	2.588	-2.585	0.1061	24.36	7.077	<0.000001
Slc15a1	No	0.60436	-0.0031	0.1398	-0.1429	0.26	0.5494	5.486	0.659301
Slc15a2	Yes	<0.000001	-0.0008857	1.874	-1.874	0.1124	16.68	9.302	<0.000001
Slc46a2	Yes	0.000009	0.001536	1.642	-1.64	0.1883	8.71	9.363	0.000026

Females

Genes	Discovery?	P value	Mean of Control	Mean of *L. reuteri*	Difference	SE of difference	t ratio	df	q value
Bdnf	No	0.212161	0.002776	0.2589	-0.2561	0.1872	1.368	7.248	0.347027
Ppp1r1b	Yes	0.007217	-0.001812	1.506	-1.508	0.3764	4.007	5.938	0.043729
Syp	No	0.918563	0.005355	-0.01642	0.02178	0.2074	0.105	9.506	0.918563
Itgam	No	0.087356	0.002625	0.8353	-0.8326	0.4015	2.074	5.535	0.262068
Il10	Yes	0.010932	-0.0005035	1.804	-1.805	0.4915	3.672	5.844	0.043729
Trem2	Yes	0.010688	0.0001815	0.6529	-0.6527	0.2047	3.188	9.23	0.043729
Mag	No	0.26027	-0.003701	0.345	-0.3487	0.2754	1.266	5.1	0.347027
Mog	No	0.628577	-0.0001115	0.09828	-0.09839	0.1935	0.5084	6.275	0.726739
Oxtr	No	0.146235	-0.002079	0.888	-0.8901	0.5231	1.702	5.3	0.29247
Slc15a1	No	0.256795	0.0006082	0.4314	-0.4308	0.3433	1.255	5.921	0.347027
Slc15a2	No	0.666177	-0.00007581	0.1877	-0.1878	0.4131	0.4546	5.697	0.726739
Slc46a2	No	0.137352	0.0001995	0.6279	-0.6277	0.3711	1.691	6.575	0.29247

Statistical analyses were performed separately for males and females. Exact P values and Benjamini–Hochberg FDR-adjusted q values are reported.
Discovery indicates genes remaining significant after Benjamini–Hochberg FDR correction applied across the full gene panel within each experimental comparison.
Difference was calculated as Control − Probiotic; negative values indicate higher expression in the probiotic group.
